# Public Awareness of Food Products, Preferences and Practices: Old Challenges and New Insights

**DOI:** 10.3390/ijerph20095691

**Published:** 2023-05-01

**Authors:** F. Xavier Medina, Francesc Fusté-Forné, Nela Filimon

**Affiliations:** 1 Unesco Chair on Food, Culture and Development, Department of Food and Nutrition, Faculty of Health Sciences, Universitat Oberta de Catalunya (UOC), Rambla del Poblenou, 156, 08035 Barcelona, Spain; 2 Department of Business, Faculty of Business and Economic Sciences, Edifici Econòmiques, c/ de la Universitat de Girona, 10, Campus Montilivi, University of Girona, 17003 Girona, Spain; francesc.fusteforne@udg.edu (F.F.-F.); nela.filimon@udg.edu (N.F.)

Food is not only a source of nutrition for humans; it also encompasses social, cultural, and psychological dynamics. Understanding of food products, preferences, and practices provides us with knowledge of historical, cultural and contemporary uses [1]. In addition, food and drink act as both identifiers and attractions of regions around the world. In this regard, there are a great variety of actors, cultures, and practices within the food value chain. This Special Issue on ‘Public Awareness of Food Products, Preferences and Practices’ discusses the protection and promotion of food, from a broad perspective, through 15 innovative research papers and systematic reviews, from both qualitative and quantitative methodological approaches.

In the first paper published in this Special Issue, Valli et al. [2] analyze the influence of people’s values and preferences on daily food choices in relation to unprocessed and processed red meat. Based on a cross-sectional mixed-methods study, they revealed that the majority of their sample was unwilling to stop, and even to reduce, their red meat consumption; this line of thinking is driven by the familial and social context of meat consumption, and by health- and non-health-related concerns about meat consumption. In a similar manner, Nestorowicz et al. [3] studied the relationship between food consumption and wellbeing, and how the diet influences levels of wellbeing. Drawing on a quantitative approach, they determined that the consumption of organic food and following a regimen such as a vegan, low-salt, or low-sugar diet results in higher levels of wellbeing; this conclusion was not only based on health, but also on pleasure and the social dimension of food.

Zorell [4] highlighted the role of influencers on individuals’ consumption decisions. Informed by a quantitative analysis, their paper shows that social media is a primary source of information about food, along with families and schools, and that this source may also lead to environmentally friendlier food consumption. Zafar et al. [5] analyzed the impact of food labels on consumers’ attitudes and intentions towards healthy and nutritional foods. Based on a quantitative study, their results show that food labels and their format not only influence consumers’ attitudes, but also their purchasing decisions. The study by Li et al. [6] focuses on food delivery services as an example of online-to-offline (O2O) commerce. The authors develop a literature review and reveal current research and industry trends.

In addition, Cipriano-Crespo et al. [7] present a qualitative ethnographic study identifying how the feeding process of people with functional diversity results in different eating situations. Their results show that influences on eating situations are mainly driven by three themes: social ghettoization and culinary loneliness; stigma, shame, feeling like a burden, and loneliness; and exclusion or self-exclusion at the dining table. Additionally, Díaz-Méndez et al. [8] analyzed the social factors that contribute to obesity as a public health problem. Based on the case of Spain, they reveal that while official statistics include socio-demographic variables, health and social variables, always understood from a social perspective, could allow the provision of more tangible support for halting obesity [9].

Muñoz et al. [10] focused on the role of food in pregnant and breastfeeding women, and its influence on both their own health and the health of their child. The paper analyzes discourses and practices in relation to the dietary intake of the participants, and shows the role of trust and mistrust in relation to food products, foods’ origins, and modes of production. Additionally, in the context of dietary intake, but specifically of nutritional interventions in surgery patients from a hospital perspective, Sole-Sedeno et al. [11] explored the impact of protein supplementation in a prehabilitation program in endometrial cancer patients undergoing laparoscopic surgery.

In the context of Poland as a particular example in Europe, Raftowicz [12] analyzed the situation of the country’s carp fishing economy. The paper alerts readers to the stagnation of a centuries-old tradition, and explains the challenges facing the development of the market because of a decrease in carp consumption by young adult consumers. On the other hand, Dudziak and Kocira [13] focused their research on the development of the organic food market. They study the barriers related to the availability of organic food and also the lack of awareness of consumers. Based on a quantitative design, the paper analyzes the determinants affecting people’s choices of organic food, these determinants being price and product labeling.

Ros-Baró et al. [14] carried out a systematic review aiming to examine the role of edible insect consumption in health outcomes, alongside its environmental impact. They reveal that edible insects are an alternative protein source that can improve human and animal nutrition, and improve the health of the planet. Later, Ros-Baró et al. [15] analyzed the factors involved in consumer acceptability of insect consumption in the Mediterranean area in a more applied way. Their quantitative study demonstrated that neophobia, social norms, familiarity, experiences of consumption, and knowledge of benefits are crucial to spread information and therefore increase insect consumption.

Dancausa Millán and Millán Vázquez de la Torre [16] analyzed the relationships between food and tourism based on quality foods endorsed by protected designations of origin (PDOs). They focused on olive oil, wine and ham. Drawing from the perspectives of gastronomic tourists, they propose strategies to deseasonalize tourism through food.

Finally, in the context of the relationship between food and tourism, Yang et al. [17] focused on the rural catering industry. They also analyzed the perspectives of consumers through social media data. Their results show that agricultural resources, safety, and a hygienic environment are important factors in the competitiveness of rural restaurants, and explain the differences between three different groups of clientele (regular customers, customers with children, and elderly customers).

This Special Issue on ‘Public Awareness of Food Products, Preferences and Practices’ discusses the conception, protection, and promotion of food from a broad perspective, analyzing food-based experiences, consumption, food cultures, social behavior related to food, and healthy and sustainable food practices. All these papers came from original and innovative international research and case studies that show food, from all perspectives, as it is: a necessary fact that straddles the biological and the social, with strong implications for our daily life. They also invite researchers and decision-makers in the field to look into future lines of research, which will span various different areas, such as artificial intelligence, advances in measuring food carbon footprint, demographic and climate change, the preservation of biodiversity, and other factors that could affect individuals’ food preferences, lifestyle, health, and wellbeing.
